# Oxygen gradient generator to improve *in vitro* modeling of ischemic stroke

**DOI:** 10.3389/fnins.2023.1110083

**Published:** 2023-03-28

**Authors:** João Santiago, Joose Kreutzer, Elsbeth Bossink, Pasi Kallio, Joost le Feber

**Affiliations:** ^1^Clinical Neurophysiology, University of Twente, Enschede, Netherlands; ^2^Micro and Nanosystems Research Group, Faculty of Medicine and Health Technology, Tampere University, Tampere, Finland; ^3^Biomedical and Environmental Sensor Systems, University of Twente, Enschede, Netherlands

**Keywords:** *in vitro* model, oxygen gradient, neuronal cultures, microelectrode arrays (MEAs), ischemic stroke, hypoxia, ischemic penumbra

## Abstract

**Introduction:**

In the core of a brain infarct, perfusion is severely impeded, and neuronal death occurs within minutes. In the penumbra, an area near the core with more remaining perfusion, cells initially remain viable, but activity is significantly reduced. In principle, the penumbra can be saved if reperfusion is established on time, making it a promising target for treatment. *In vitro* models with cultured neurons on microelectrode arrays (MEAs) provide a useful tool to investigate how ischemic stroke affects neuronal functioning. These models tend to be uniform, focusing on the isolated penumbra, and typically lack adjacent regions such as a core and unaffected regions (normal perfusion). However, processes in these regions may affect neuronal functioning and survival in the penumbra.

**Materials and methods:**

Here, we designed, fabricated, and characterized a cytocompatible device that generates an oxygen gradient across *in vitro* neuronal cultures to expose cells to hypoxia of various depths from near anoxia to near normoxia. This marks a step in the path to mimic core, penumbra, and healthy tissue, and will facilitate better *in vitro* modeling of ischemic stroke.

**Results:**

The generator forms a stable and reproducible gradient within 30 min. Oxygen concentrations at the extremes are adjustable in a physiologically relevant range. Application of the generator did not negatively affect electrophysiological recordings or the viability of cultures, thus confirming the cytocompatibility of the device.

**Discussion:**

The developed device is able to impose an oxygen gradient on neuronal cultures and may enrich *in vitro* stroke models.

## Introduction

Ischemic stroke is the most common form of stroke and is caused by the blockage of one of the brain arteries, leading to the reduction of blood flow (Lakhan et al., 2009). This limits the availability of oxygen and glucose and can lead to cell damage or death. The environment of an ischemic stroke is complex, extending from the core, where perfusion levels are below 10 mL/100 g/min (severe ischemia) and damage tends to be irreversible, to the penumbra (perfusion levels between 14 and 35 mL/100 g/min), which is salvageable if there is reperfusion in time, and to healthy tissue with undisturbed perfusion. The initial reversible character of damage in the penumbra, and the relatively long timescale at which progression to irreversible damage occurs, make it a suitable target for clinical intervention (Hakim, 1987). Consequently, much research has focused on the ischemic penumbra trying to mimic it and evaluate possible treatment to improve recovery.

Various *in vitro* studies have been conducted that focused on stroke, based on brain slices or dissociated cultures (Holloway and Gavins, 2016). Many focused on processes that occur in the infarct core, and studies that have been conducted to simulate the ischemic penumbra generally assumed a uniform model in which neuronal cultures were exposed to hypoxia at a constant and controlled depth (Le Feber et al., 2016; Muzzi et al., 2017; Pires Monteiro et al., 2021). The use of microelectrode arrays (MEAs) makes it possible to measure their activity throughout experiments. Such models have shown that hypoxia quickly induced synaptic failure, and the resulting reduced activity has been hypothesized to trigger apoptosis in the penumbra (Taxis Di Bordonia et al., 2022). In support of this hypothesis, they also showed that mild stimulation may be beneficial for cell survival and recovery of neuronal activity following reoxygenation (Khazipov et al., 1995; Sun et al., 2002; Muzzi et al., 2017).

However, these models do not include the spatial differences that are typical for *in vivo* stroke, with varying ischemic depths. Consequently, they do not capture possible interaction between the differently affected areas. For example, glutamate or potassium ions released from necrotic cells in the core or persisting activity in healthy tissue may activate neurons in the penumbra, which is plausible to affect the beneficial effect of therapeutic stimulation, or the optimum stimulation parameters to improve neuronal survival. Thus, it is important to study the entire environment, including regions of varying hypoxic/ischemic depth.

Several studies have reported on the design of oxygen gradient generators to be used with tumor cells (Uchida et al., 2013; Khan et al., 2017; Orcheston-Findlay et al., 2020), brain slices (Mauleon et al., 2012), 3D cultures in hydrogel (Boyce et al., 2018), or endothelial cells (Rexius-Hall et al., 2017). However, none of these devices facilitate electrophysiological recording, as they did not contain integrated electrodes, and were not designed to be compatible with commercially available MEAs.

Here, we design, fabricate, and characterize a new device that generates an oxygen gradient for *in vitro* neuronal cultures to be used in MEA-based *in vitro* stroke models. The oxygen gradient generator creates an oxygen gradient across an area with dimensions that match the electrode area of commercially available MEAs. The generator ensures a stable, reproducible oxygen gradient between set maximum (< normoxia) and minimum (> anoxia) oxygen concentrations. Finally, the generator is demonstrated to be compatible with cells and technically validated to facilitate undisturbed electrophysiological recordings.

## Materials and methods

### Recording setup

**FIGURE 1:**
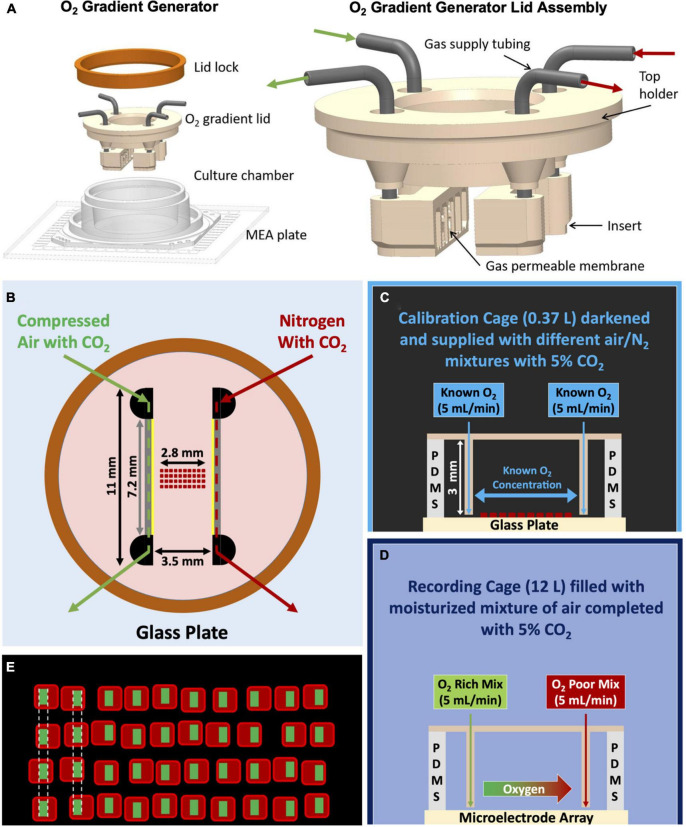
Various schematics of the oxygen gradient generator and the setup. **(A)** 3D model of the device. After closing the top of the culture well (MEA with the culture chamber) with the gradient lid, it is sealed with the lid lock (orange ring). The lid assembly (on the right) consist of a top holder, two inserts and 19G stainless-steel connectors. The inserts are facing each other and each one has attached a gas permeable silicone membrane. **(B)** Top view of the device sealed with the ring, indicating the dimensions of the device and the placement of the oxygen sensors grid. Membranes through which gas exchange takes place (7.2 × 3 mm) are indicated in yellow. The lower border of the membranes was ∼0.2 mm above the surface of the MEA. **(C)** Representation of the complete setup for calibration with the calibration cage (smaller and darkened), the grid of sensors which was exposed to gas mixtures of known O_2_ concentrations, and a cross-sectional view of the glass plate, silicone culture chamber and lid assembly. **(D)** Representation of the complete setup for cell experiments in the recording cage (bigger and not darkened), the gas mixtures supplied to the generator (green: compressed air with CO_2_; red: N_2_ with CO_2_), and a cross-sectional view of the MEA, silicone culture chamber and lid assembly. **(E)** Representation of the grid of sensors and the definition of the ROIs for the calculation of the red phosphorescence intensity and estimation of oxygen concentration.

We obtained cell cultures from brain cortices of newborn Wistar rats (male and female). All surgical and experimental procedures complied with Dutch and European laws and guidelines (AVD110002016802). We treated the cortices with trypsin and then dissociated them by titration before seeding on multielectrode arrays (MEAs, Multichannel Systems, Reutlingen, Germany) precoated using a 50 μg/mL solution of polyethylene imine (PEI). We attached a silicone-made culture chamber on top of the MEA to create a 1 mL well to contain culture medium (Figure 1A). Cultures were kept in R12H medium (Romijn et al., 1984). The medium contained a color-based pH indicator, which presents a red toned color under normal culturing conditions and turns pink at increased pH, usually reflecting decreased CO_2_. Cultures were grown in an incubator under standard conditions of 37°C, high humidity and 5% CO_2_ for at least 3 weeks before the start of experiments. Half of the medium was refreshed twice a week.

For experiments, MEAs were transferred to a temperature-controlled recording setup, as described in Stegenga et al. (2008). This setup was placed inside a gas-controlled chamber of 12 L which also worked as a Faraday cage to maximize the quality of the readings and is referred to as the recording cage (Figure 1D). Continuous flow (2 L/min) of a moisturized mixture of air and N_2_, complemented with 5% CO_2_, was supplied into the recording cage. We recorded electrophysiological activity using custom software driving a PCI-6023E data-acquisition card (National Instruments, Austin, TX, USA) at a sample frequency of 16 kHz per channel. Action potentials were detected using a threshold crossing algorithm. For all electrodes, the RMS noise level was continuously estimated and a detection threshold was set at 5.5 times this level (le Feber et al., 2007). We stored timestamps and channel numbers of detected events, as well as 6 ms of wave shape and the estimated noise level at that electrode at the time of the event. We then used the shapes of putative action potentials for off-line artifact detection using an algorithm adopted from Wagenaar et al. (2005). False positive detections were counted and removed before analysis.

We designed the gradient generator to be used with two different MEAs layouts of 60 electrodes: the 60MEA200/30iR-Ti-w/o or the 60-4QMEA1000iR-Ti-w/o (both Multichannel Systems, Germany). The first one contains an 8 by 8 grid of TiN electrodes (Ø: 30 μm) spaced 200 μm from each other (spanning a recording area of 1.4 mm in length) and the second one has four quadrants with 13 electrodes each, and a central line with seven electrodes, covering a recording range of 2.6 mm in length. Data presented here, were collected using 60MEA200/30iR-Ti-w/o.

### The oxygen gradient generator

The oxygen gradient generator is a device that forms an oxygen gradient between two inserts through which gas mixtures of known concentrations are flowing. In this study, the oxygen gradient was formed across neuronal cell culture network on the electrode area of commercially available MEAs. The generator consists of three parts: a silicone culture chamber, a tailored O_2_ gradient lid assembly to establish the oxygen gradient inside the culture chamber and a metal lid lock (Figure 1A). The culture chamber is attached to the MEA to allow cell culturing in 1 mL culture medium and continuous MEA recordings under physiological conditions (Metsälä et al., 2018; Häkli et al., 2021, 2022). The tailored lid assembly consists of a top holder, two inserts and connection tubing (Figure 1A).

The top holder and inserts are 3D printed with biocompatible dental resin using a Form3 printer (Formlabs Inc., USA). A 20 μm thick silicone membrane (Silpuran Film 2030, Wacker Chemie AG, Germany) is glued to the face of each insert using uncured liquid PDMS and let to cure. Then, the membrane is gently cut around the face of the insert with a tiny scalpel. The thin silicone membrane allows rapid and efficient gas exchange (Tornberg et al., 2022) from the insert to the cell culture. To enhance transparency through the lid and visibility of cells, we printed the top holder as a hollow cylinder and attached a thin (0.17 mm) Ø 12 mm cover glass to the bottom of the holder. We connected the two inserts to the top holder with stainless steel tubing (19G) and fixed the inserts facing each other with a distance of 3.5 mm (Figure 1B).

There was a 3.5 mm distance between the membranes (Figure 1B) in which the oxygen gradient was formed on the bottom of the MEA plate. Finally, we sealed the lid assembly against the silicone culture chamber with a lid lock (orange ring in Figure 1A) to minimize air leakage and evaporation. The inserts are designed to receive a gas flow of 5 mL/min. Pressures associated with this flow through the inserts are low enough to avoid any deformation of the membrane.

### Flow rates: Setup and validation

The flow rate of 5 mL/min in each insert was achieved using three mass-flow controllers (Vogtlin Instruments, Switzerland; compressed air, max 5 L/min; N_2_, max 5 L/min; CO_2_, max 0.5 L/min) followed by a bypass assembly for both inserts. Bypasses reduce the output flow generated by the flow controllers to the required 5 mL/min flow for the inserts (Figures 1C, D).

The bypass assembly was constructed from two parallel pneumatic resistors, the larger (R_1_) leading to an insert of the gradient generator and the smaller (R_2_) to open air. As R_1_ was much higher than the resistance of the insert channel, the flow through the insert was determined by the following equation: *Q*_*channel*_ = *R*_2_/(*R*_1_ + *R*_2_)⋅Q_*flow controller*_. We verified the output flow rates of both bypasses before every experiment, by measuring the cumulative gas volume in a submerged measuring cup during 5 min.

### Oxygen sensing: Principle, design, and fabrication

We used small Platinum Tetrakis (Pentafluorophenyl) Porphyrin (PtTFPP) sensors (squares of 220 ± 20 μm side) to enable oxygen measurement with sufficient spatial resolution. This metalloporphyrin is a photoluminescent molecule whose phosphorescence is quenched by ambient oxygen, proportionally to the oxygen concentration (Välimäki et al., 2020). As so, the phosphorescence intensity increases as the level of oxygen decreases (Lee and Okura, 1997).

Polystyrene (PS, MW 280,000, Sigma-Aldrich) was dissolved in chloroform (7%w/v) and subsequently add to PtTFPP (1%w/v, Frontier Scientific) (Bossink et al., 2020). We casted this mixture on an in-house made aluminum mold. The chloroform is left to evaporate for at least 24 h, whereafter the PtTFPP patch can be removed from the mold.

From one of these patches, a grid of sensors was created by manually cutting 40 square sensors and placing them in a 10 × 4 matrix (pitch ∼50 μm) under the microscope (Figure 1E). To keep the sensors in their places, and to attach them to the bottom of the MEA, the grid was embedded in polydimethylsiloxane (PDMS) (Sylgard 184, Dow Corning Inc., USA). First, we carefully poured a 170 μm thick bottom layer of PDMS (curing agent:base = 1:10% w/w) in a glass mold (24 × 10 mm) and pre-cured it for 30 min at 65°C. After placement of the sensors on top of the pre-cured PDMS, we covered them with another layer of PDMS (curing agent:base = 1:7.5% w/w). After vacuuming to remove air bubbles (for 15 min in cycles of vacuum and no vacuum to force air bubbles to burst), the assembled strip was cured for 15 min at 65°C. Different curing agent:base ratios were used to ensure that the first layer remained sufficiently flexible.

The grid of sensors covers a total length of approximately 2.8 mm, slightly smaller than the 3.5 mm distance between the inserts of the generator, to facilitate manual placement between the inserts while minimizing the risk of touching and possibly damaging the thin PDMS membranes of the generator.

### Oxygen sensing: Calibration

We placed the generator on top of a culture chamber attached to a glass bottom plate, inside a gas-controlled chamber with a volume of 0.37 L, referred to as the calibration cage (Figure 1C). We darkened the chamber to avoid interference from external light sources with the luminescence intensity measurement. We then supplied gas mixtures of controlled composition of compressed air and N_2_ to the calibration cage to create a controlled O_2_ concentration inside this environment.

We filled the culture chamber with culture medium and supplied six different gas mixtures both through the two inserts of the generator and to the chamber. We used a commercial oxygen sensor (NeoFox, Ocean Optics, Duiven, The Netherlands) to verify that oxygen levels inside the calibration cage showed no strong variations during the time of the calibration process (validation of oxygen steadiness). Air:N_2_ ratios of the mixture were 0:100, 20:80, 40:60, 60:40, 80:20, and 100:0. For the calibration, the gas mixtures were not supplemented with CO_2_ to achieve any specific oxygen concentration between 21 and 0%.

Every change of oxygen concentration was followed by a 1-h stabilization period. Stability of readings was verified by fitting a trend line to the values of phosphorescence intensity in a 15-min moving window.

We considered phosphorescence values for a given oxygen concentration to be stable when the slope of the trend line was smaller than 1% O_2_/15 min. If such threshold was not reached, readings were not taken into account. After reaching a stable intensity, the oxygen concentration was considered equal to the concentration in the supplied gas mixture, and images were obtained every 15 min for another 120–180 min. All images were taken at 20× magnification using a Nikon Eclipse 50i fluorescence Microscope and a Nikon DS-Fi1 digital camera.

We set regions of interest (ROIs) for each sensor (Figure 1E) following two criteria: ROIs did not include the edges to avoid errors due to scattering, and ROIs were perfectly aligned in lines perpendicular to the intended oxygen gradient. For each oxygen concentration, red phosphorescence intensities (RPI) were averaged per column of aligned ROIs and used to construct calibration curves between phosphorescence intensity and oxygen concentration.

We used a Stern–Volmer derived approach for calibration, with RPI_0_/RPI as independent variable [RPI is the red phosphorescence intensity for a given oxygen concentration and RPI_0_ is the red intensity with no oxygen (Gehlen, 2020)]. The calibration process was conducted before each gradient measurement and the sensor grid was not moved between the calibration and the gradient experiments to minimize possible calibration errors related to the exact placement and illumination of the sensors.

To evaluate whether the usage of culture medium with a colored pH indicator affected the calibration, an additional calibration curve was determined in water following the same protocol.

### Oxygen gradient

Oxygen gradients were established by application of different gas mixtures to the inserts of the generator. Here we used 95% compressed air + 5% CO_2_ for one insert and 95% N_2_ + 5% CO_2_ for the other, and measured oxygen concentrations in the medium between the inserts. We defined the direction from one insert to the other as longitudinal, and the transverse direction as parallel to the inserts. We measured whether there was a transverse component in the gradient, which might affect oxygen concentrations in the area where cells are seeded (typically a ∼2 × 2 mm square in the center). To do so, we repeated measurements with inverted direction of gas flow in the inserts. We compared readings after 1 h of flow in either direction (to allow for stable readings as before). These readings were considered stable if changes in oxygen concentration after this point were < 1% O_2_ per 15 min.

In one experiment, we evaluated whether the oxygen concentrations measured between the inserts in the culture chamber was independent from the composition of gasses in the calibration cage (surrounding the culture chamber). To do this, we changed the gas mixture inside the calibration cage from N_2_ to air (with N_2_ being supplied to the culture medium through both inserts), while oxygen concentrations between the inserts were continuously measured. This experiment was performed using an earlier version of the grid with only seven columns of sensors.

All oxygen measurements were done on glass bottoms, electrophysiology experiments in the following section were done on MEAs, without simultaneous oxygen sensing.

### Compatibility with electrophysiological recordings

We used four different configurations to determine whether the oxygen gradient generator affected the noise recorded by the MEA electrodes, whether flow through the inserts induced movement artifacts, and whether the generator affected neuronal viability. All configurations were applied for 1 h except the “Generator Gradient” configuration, which lasted 2 h for proper gradient establishment.

1. **Conventional Lid:** Culture chamber with the culture in R12 medium on the MEA closed with a conventional lid consisting of a Teflon ring (Multi Channel Systems, Reutlingen, Germany) with O_2_ and CO_2_ permeable foil (ALA-Scientific, Germany).
2. **Generator Lid-off:** Conventional lid replaced by the O_2_ gradient lid (no gas flow through the inserts).
3. **Generator Normoxia:** Both inserts supplied with 5 mL/min 95% compressed air with 5% CO_2_.
4. **Generator Gradient:** Gas flow through one insert altered to 95% N_2_ with 5% CO_2_ (establish a gradient).

In each configuration we registered the following readouts: (1) the number of electrodes that recorded at least five action potentials per minute after artifact removal (active electrodes), (2) the number of removed artifacts for each active electrode (expressed as a fraction of the total number of putative detections), (3) the RMS value of the noise captured by the electrodes (averaged across all active electrodes), (4) the mean firing rates per active electrode (meaning the average number of action potentials per minute), and (5) the average shape of detected action potentials (similarities evaluated by correlation coefficients as well as amplitude variations). Additionally, as activity patterns of healthy normoxic cultures typically contain network bursts (periods of synchronized firing at many electrodes), we qualitatively evaluated whether activity patterns contained network bursts by visual inspection of raster plots (burstiness evaluated as present or absent).

Comparison between configurations “Conventional Lid” and “Generator Lid-off” make it possible to see if the gradient device affects recorded noise. Changes between configurations “Generator Lid-off” and “Generator Normoxia” indicate whether gas flow through the inserts induced movement artifacts. Differences between configurations “Generator Normoxia” and “Generator Gradient” were not deeply evaluated in this study but further analysis of differences between them would allow to study the effects of the oxygen gradient on neuronal functioning.

Finally, activity patterns were evaluated again 1–3 days after the experiment, in terms of firing rates, the number of active electrodes and the presence of network bursts. These readouts were obtained in a “Conventional Lid” configuration and compared to the ones recorded prior to exposure to the gradient, to determine whether the usage of the generator had affected culture viability.

### Statistical analysis

Group values were tested for normality using a Shapiro–Wilk test. If normally distributed, data are presented as mean ± SD, and paired *t*-Tests (2-sided) were used to assess the significance of differences. Otherwise, median values are shown with error bars indicating the 25% to 75% interquartile range (IQR), and Wilcoxon signed rank tests were used (non-parametric variant of a paired *t*-test). Bonferroni correction was applied for multiple comparisons. *P* < 0.05 was considered to indicate significant differences.

## Results

### Calibration of oxygen sensors

**FIGURE 2:**
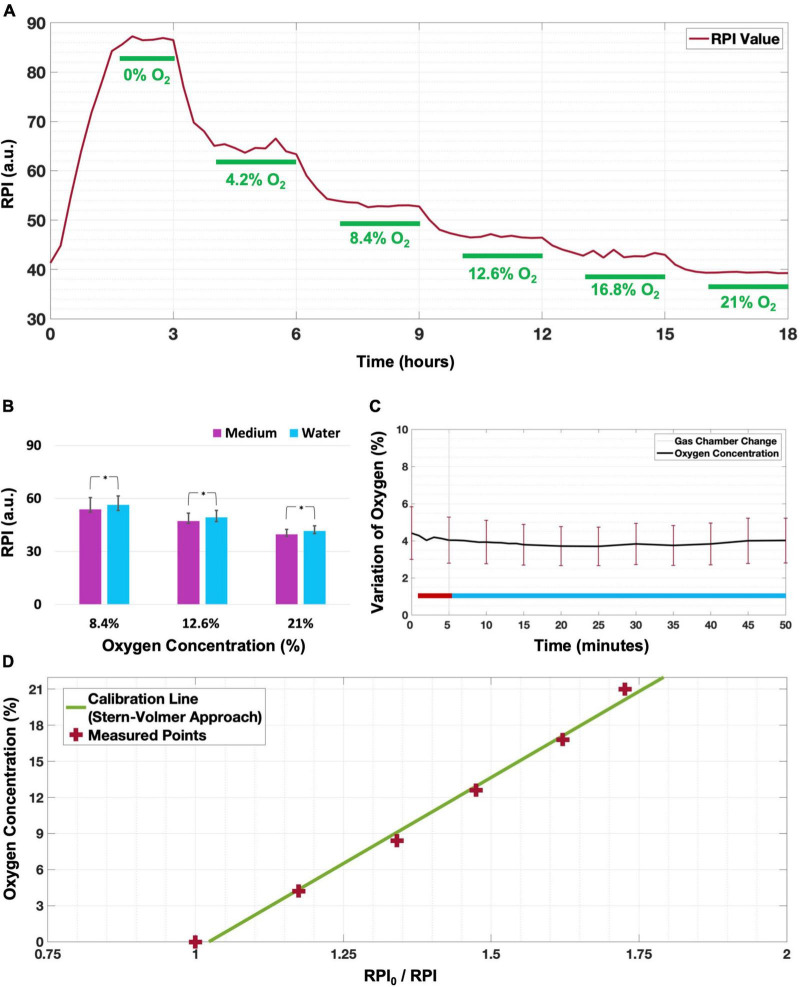
Calibration of the oxygen sensors. **(A)** Example of stepwise phosphorescence intensity changes observed at one of the sensors when supplying both channels of the gradient generator with the six known gas mixtures, indicated in each of the steps that were considered stable (marked as green). **(B)** At all oxygen concentrations, phosphorescence (median ± 25–75% quartiles) was slightly but significantly lower when measured in culture medium than in water (*p < 0.001 for a sample of *n* = 35 electrodes). **(C)** When exposing all sensors to N_2_ through the inserts while the gas supplied to the calibration cage was switched from N_2_ (red line period) to air (blue line period), changes were less than 1% O_2_ during a period of 45 min (*n* = 40 sensors; mean ± SD). **(D)** Example of Stern-Volmer equation derived calibration for one sensor with *R*^2^ = 0.997.

For calibration, we placed the array of sensors inside a well with culture medium and exposed it to six different oxygen concentrations (Figure 2A). This process was repeated to obtain two independent calibrations, as well as and one calibration before each gradient experiment, to mitigate possible effects of the exact sensor placement. No air bubbles were observed during the calibration process. In two calibrations, not all sensors reached stable readings in the first step, which was excluded in these calibrations. All other oxygen concentration steps reached stable readings within 1 h (Figure 2A) in all calibrations. The average phosphorescence intensity per oxygen concentration was used to fit a calibration curve for each sensor (Figure 2D shows an example for one of the sensors). Stern-Volmer equations were fitted for all sensors and averaged, yielding:


(1) [O]2(%)=[27.4±2.7]⋅RPI/0RPI-[28.2±2.7]

where RPI_0_/RPI is the quotient of the red phosphorescence intensities (RPI) in an oxygen free environment (I_0_) and RPI at the actual oxygen concentration (I). Repeated calibrations yielded parameters with slight differences, but with very similar coefficient of variation (= SD/mean).

Repetition of the calibration protocol in water yielded slightly but significantly higher RPI (+ 1.12 ± 1.00 arbitrary units), indicating that the color of the medium hardly affected the readout (Figure 2B; Wilcoxon signed rank *p* < 0.001).

After changing the surrounding gas mixture from 95% N_2_ + 5% CO_2_ to 95% air + 5% CO_2_, the oxygen level changed less than 1% of O_2_ during a period of 45 min after the change (Figure 2C), confirming that the oxygen concentration between the inserts was independent from the chamber’s gas composition.

### Oxygen gradient

**FIGURE 3:**
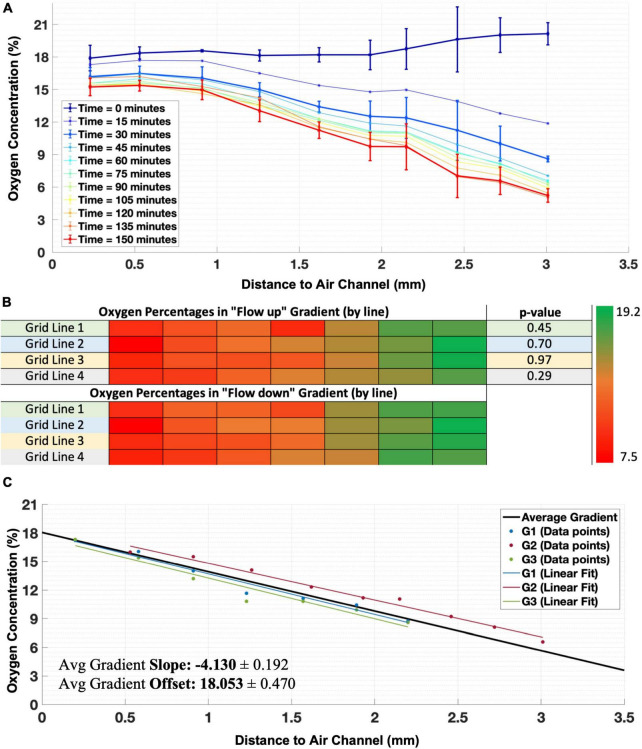
Establishment of the oxygen gradient. **(A)** Example of time course of establishment of the oxygen gradient in one illustrative experiment, showing stability and linearity of the gradient after 30 min. Error bars indicate SD and refer to differences between sensors in the same column perpendicular to the gradient (*n* = 4 sensors per column). For clarity error bars are shown only for *t* = 0, *t* = 30 min, and *t* = 150 min, other curves had error bars of comparable magnitude. **(B)** Changing the direction of flow through the inserts did not significantly change the readout values of sensors in lines of the grid (direction of the gradient), showing that there was no transverse component. **(C)** Real gradients observed in three experiments (shown for the measurable length using the grid of sensors in each experiment) which were averaged and extrapolated for the full gap between the inserts (Average Gradient, traced in black) to obtain the mean slope and offset for the generalization of the oxygen gradient produced by the generator.

We applied a mixture of 95% compressed air with 5% CO_2_ to one insert and 95% N_2_ with 5% CO_2_ to the other and characterized the formed gradient in three independent experiments. A clear oxygen gradient appeared within 30 min, which became slightly steeper in the following 30 min and then remained stable for at least 3 h (Figure 3A).

In one experiment, we inverted the direction of gas flow through the inserts. Phosphorescence intensity measurements after 1 h of gradient forming showed no significant differences between oxygen concentrations created by flows in either direction (paired *t*-test: 0.29 < *p* < 0.97 for all sensors, Figure 3B).

Gradients were very similar in three independent experiments, with (O_2_) ranging from approximately 4–18% oxygen across the 2.8 mm wide area covered by the sensors (Figure 3C). The usage of the oxygen gradient generator inside the culture chamber did not induce visible air bubbles in the membranes or in the culture medium.

### Electrophysiological recordings

We used two cultures to evaluate the cytocompatibility of the generator, as well as possible interference of the generator with electrophysiological recording. Combining results from both experiments, 41 electrodes (20 + 21 in the two cultures) remained active throughout the four different configurations. Seven electrodes (2 + 5) that were active in the “Conventional Lid” setup became inactive during the following phases of the experiment (three of which showed already less than 15 action potentials per minute during the “Conventional Lid” phase). In both cultures the location of these electrodes seemed uncorrelated to the different time courses of local oxygen concentrations. Eight electrodes (5 + 3) became inactive during the “Generator Gradient” phase. These electrodes tended to be on the low end of the oxygen gradient. All eight regained their activity upon restoration of normoxia. Table 1 summarizes the main readouts extracted from electrophysiological recordings.

**TABLE 1:** Main electrophysiology readouts evaluated for each configuration.

Configuration	Active electrodes	Artifacts (%)	RMS noise (μ V)	Firing rate (spikes/electrode/min)
1. Conventional lid	56	26.7	4.4 [3.8–5.1]	67 [28–154]
2. Generator lid-off	49	26.6	4.8 [4.2–5.2]	39 [20–100]
3. Generator normoxia (pre-gradient)	49	23.8	4.7 [4.2–5.2]	33 [22–70]
4. Generator gradient	41	12.1	4.7 [4.0–5.2]	16 [8–45]
5. Generator normoxia (post-gradient)	49	18.2	4.1 [3.6–5.1]	39 [13–129]
Follow up (conventional lid)	34	27.7	Not evaluated	61 [52–186]

Activity noise and artifacts were subsequently determined in several configurations: 1–conventional lid, 2–generator lid with no gas flow through the inserts; 3–generator lid with compressed air + 5% CO_2_ through both inserts; 4–generator lid with compressed air + 5% CO_2_ in one insert and N_2_ + 5% CO_2_ in the other; and 5–generator lid with compressed air + 5% CO_2_ through both inserts. Active electrodes and percentage artifacts show cumulative results from 2 independent experiments. RMS noise and firing rate are shown as median and [range].

### Effect of inserting the generator

**FIGURE 4:**
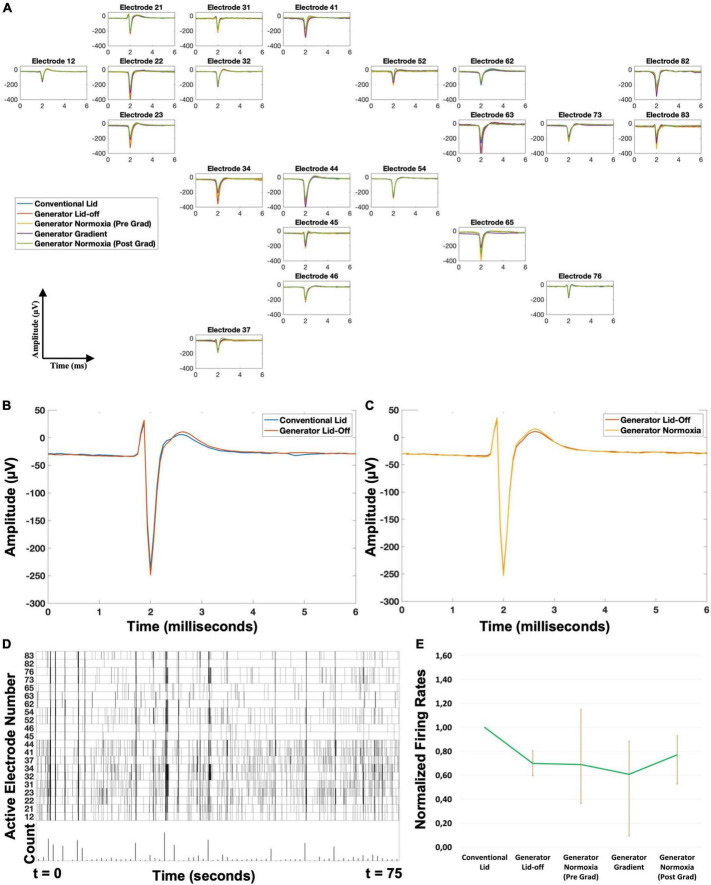
Electrophysiological recording **(A)** example of recorded action potentials during the various phases in one experiment, where 21 of the 60 electrodes recorded activity. Position in the figure represents the position of recording electrodes in the Microelectrode Array. Mean waveform of action potentials (shape and amplitude) did not significantly change between phases. **(B)** Close up of a representative electrode showing the similarities in mean waveform between the phases “Conventional Lid” and “Generator Lid-off.” **(C)** Close up of a representative electrode showing the similarities in mean waveform between the phases “Generator Lid -off” and “Generator Normoxia.” **(D)** Example of a raster plot (only active electrodes), with instances of network bursts. **(E)** Evolution of the mean firing rates per minute during the recordings, normalized to “Conventional Lid” values, showing that there is a decrease in the “Generator Lid-off” and “Generator Gradient” phases, both compensated when oxygen flow through the channels is restored (error bars indicate 25–75% quartiles; *n* = 41 active electrodes from both experiments combined). All phases lasted 1 h, except “Generator Gradient,” which lasted 2 h.

Mean waveforms per electrode showed no differences in shape between the “Conventional Lid” and “Generator Lid-off” configurations (Figures 4A, B) with correlations of 0.99 ± 0.01 for all electrodes. The amplitude of the waveforms showed only minor fluctuations (<6% of the mean amplitude for all electrodes). There was a negative shift on the distribution of firing frequencies per electrode between the “Conventional Lid” and “Generator Lid-off” with a median decrease of 30% and IQR = 19–40% (Wilcoxon test: *p* < 0.005), as shown in Figure 4E. The artifact detection algorithm showed no considerable difference in the fraction of detected artifacts between these configurations (26.7 vs. 26.6%, more than half of the detections were due to bigger peaks in vicinity). Estimated noise levels slightly increased when the gradient generator was inserted without gas flow (median of 4.4 μV with IQR = 3.8–5.1 μV vs. median of 4.8 μV with IQR = 4.2–5.2 μV; Wilcoxon test: *p* < 0.001).

### Possible movement artifacts

Differences in readouts between configurations “Generator Lid-off” and “Generator Normoxia” were evaluated (Figure 4A, C). Once again, mean waveforms per electrode showed no differences in shape with average correlations of 0.99 ± 0.02 and amplitude differences were < 1% for all electrodes. No considerable changes in the percentage of artifacts detected were observed when a flow was directed through the gas channels (26.6 vs. 23.8%, once again, more than half of the detections were due to bigger peaks in vicinity). Firing rates per electrode significantly changed with a median increase of 21% and IQR = −12 to 73% from “Generator Lid-off” to “Generator Normoxia” (Wilcoxon paired test: *p* < 0.001), as shown in Figure 4E. Onset of a gas flow through the inserts did not affect noise estimates at the electrodes (median of 4.8 μV with IQR = 4.2–5.2 μV vs. median of 4.7 μV with IQR = 4.2–5.1 μV; Wilcoxon paired test: *p* > 0.5).

### Oxygen gradient’s effect on cells

Despite the small sample size, on average, firing rates decreased significantly when the gradient was established, (Table 1, Wilcoxon test: *p* < 0.001), but there was large variability between electrodes, with no clear correlation between the magnitude of activity decrease and electrode location. Upon restoration of normoxia, activity at most electrodes recovered (Table 1, Wilcoxon paired test: *p* < 0.005). Noise levels remained in the same range, even though they showed slight changes in distribution between the oxygen gradient (median of 4.7 μV with IQR = 4.0–5.2 μV) and subsequent reoxygenation (median of 4.1 μV with IQR = 3.6–5.1 μV; Wilcoxon paired test: *p* < 0.01).

### Compatibility with cells

Activity patterns recorded in two cultures on the day of the experiment, as well as during follow-up (1–3 days later), all included network bursts (Figure 4D). From the total of 41 active electrodes during both experiments, 34 were still active in the follow-up recording. The firing rates for only these electrodes did not show significant differences between recordings made using the “Conventional Lid” configuration before gradient exposure and 1–3 days later (spikes/min rates with a median of 87.2 with IQR = 52.4 – 185.6 vs. a median of 61.8 with IQR = 13.7–169.3; Wilcoxon signed rank: *p* > 0.5).

## Discussion

### Main findings

In this work, an oxygen gradient generator was designed, constructed, and validated, establishing a robust oxygen gradient, extending from approximately 4–18% across neuronal networks cultured on MEAs. The gradient was established within 30 min and remained stable for at least 3 h. The usage of the generator did not disturb electrophysiological recording of neuronal activity, and cultures remained viable and active.

### Other approaches to create oxygen gradients

Some other attempts have been made to impose an oxygen gradient on cell cultures. Rexius-Hall et al. (2017) established a stable gradient within 30 min in cultures of endothelial cells, similar to the gradient that was established in the current study. Their design requires a constant pressure of 5 psi (34.5 kPa), whereas the much lower pressure across the membrane in the device developed by us (about 0.1 kPa) makes it less prone to bubble formation on the culture side of the thin membrane.

Mauleon et al. (2012) aimed to submit various regions of brain slices to different oxygen concentrations. They created microfluidic add-ons with multiple microchannels, allowing for different gas mixtures to be supplied to different areas, leading to areas with clearly different oxygen concentrations. In contrast, in the current device a continuous gradient is established over a gap between two channels that conducted different gas mixtures.

Boyce et al. (2018) developed a device to impose an oxygen gradient on 3D cultures suspended in a hydrogel, and several devices have been developed to create and measure oxygen gradients in tumors, with gas channels underneath the culture wells (Uchida et al., 2013) or perfusion with pre-gassed medium (Khan et al., 2017; Orcheston-Findlay et al., 2020). Each device was made to match requirements specific to those studies, resulting in various designs, but none of them contains integrated electrodes for electrophysiological recording, and they cannot be used with commercially available MEAs. The oxygen gradient generator presented in the current work extends these approaches by adding the possibility to record electrophysiological activity, a crucial readout to study neuronal functioning.

### Validation of the calibration

Periods of 1 h were usually sufficient to reach a stable O_2_ concentration (fitted slope smaller than 1% O_2_/15 min) after gas supply changes. However, for the largest changes, from “normoxia” to “anoxia” (∼21% O_2_ to 0% O_2_), 3 h was often not enough to completely deplete the medium from oxygen. Consequently, stability was not reached, and corresponding values were not used for calibration.

Differences between calibration curves obtained for different sensors were very small, resulting in relatively small standard deviations of the fitted parameters (Eq. 1), showing the robustness of the approach.

The composition of the gas mixture surrounding the culture chamber did not affect oxygen readings between the two inserts of the gradient generator (oxygen changed less than 1% over 45 min), which confirms that local oxygen concentrations between the two inserts were fully determined by the gas mixtures flowing through the inserts. This highlights the independency of the oxygen gradient between the inserts from the oxygen concentration inside the chamber. The relatively large error bars in Figure 2C reflect differences in oxygen readings from the various sensors and do not indicate variations in oxygen dependent on the composition of the chamber.

Although gas exchange through the silicone walls of the culture chamber is normally sufficient to maintain healthy and active cultures (Häkli et al., 2021; Gaballah et al., 2022), the gradient generator is able to actively reduce the oxygen concentration near the N_2_ insert in an oxygen rich environment. In addition, the gradient generator is able to create near normoxic conditions near the compressed air insert, even in a severely hypoxic environment. This probably reflects the fact that the walls of the culture chamber were ∼100 times thicker than the membranes of the generator, which makes the time needed for oxygen to diffuse (in one dimension) through the culture chamber walls ∼10^4^ times longer (Boal, 2012). Consequently, cells are exposed to oxygen concentrations that are fully controlled by the generator, and not by environmental oxygen concentration, which minimizes its vulnerability to environmental fluctuations.

The color of the culture medium did influence the detected fluorescence intensity and the calibration as variations of one unit of phosphorescence translate into variations of 0.1% O_2_. The slight differences between water and culture medium are probably due to the culture medium including the pH-indicator phenol red, which may have affected RPIs. This justifies conducting calibrations in culture medium since it makes the readings robust against changes in color due to pH variations.

### Temporal establishment of the gradient

The oxygen gradient was established within 30 min and remained stable after that. Apoptosis in the penumbra typically occurs after 6–24 h (Taxis Di Bordonia et al., 2022), which is an order of magnitude longer than the time needed to establish the gradient. As so, establishment of a stable oxygen gradient in 30 min seems suitable for adequate modeling of the ischemic penumbra.

### Extrapolation of the gradient

The oxygen gradient as measured by the sensors did not cover the entire 3.5 mm between the two channels of the gradient generator because of small distances between these and the sensors. Applying a linear fit to the measured gradient, the full gradient can be estimated as:


(2) O(%)2=[-4.1±0.2]⋅d+a⁢i⁢ri⁢n⁢s⁢e⁢r⁢t[18.1±0.5]

where d_*air insert*_ equals the distance (in mm) to the insert that conducted air (Figure 3C).

Linear extrapolation yields a gradient from estimated oxygen concentrations between a minimum of 2.6% near the nitrogen insert and a maximum of 18.7% close to the compressed air insert. Considering that the gas mixtures are supplied with 5% CO_2_, the maximum oxygen concentration would be around 20%. Moreover, the high humidity further reduces the maximum oxygen concentration to an estimated 18.6% in humid air with 5% CO_2_ (Place et al., 2017). Thus, the estimated oxygen concentration at the high end of the gradient approaches the theoretical maximum. At the low end of the gradient, the oxygen concentration remains higher than zero. This might be related to relatively slow diffusion of oxygen toward the N_2_ insert (and thus decreased oxygen uptake by the insert) when the driving force, the partial oxygen pressure difference toward the insert, decreases.

It might be hypothesized that higher flow rates through the inserts might produce steeper gradients, with extremes closer to 0 and 21%. However, this increases the risk of rupture of the membrane or possible bubble formation on the inner surface of the membrane due to the increase of the pressure inside the insert. Furthermore, we believe that increasing the flow rate would most likely not have the desired effect. In fact, considering the dimensions of the inserts and flow rate through the insert it is possible to infer that in the volume between the inserts a gas refreshment rate of ∼20 times per minute is established, which should be enough to build up the oxygen gradient. It seems more likely that the slightly lower range measured was related to other factors, like the use of a linear fitting for the calibration, which may lead to slight but systematic underestimation of high concentrations, and overestimation of low concentrations (Figure 2D), and thus to a lower total range.

### Noise and artifacts

The amplitudes of action potentials as measured by MEAs tend to be in the order of tens of μV (Hottowy et al., 2012), which should be detected in the presence of noise. This is usually done using a set detection threshold based on the estimated noise level, which makes detection of activity sensitive to changing noise levels at the electrodes. MEA recordings are also prone to movement artifacts (Shaw et al., 1999). The flow through the inserts is quite close to the recording sites and might in principle lead to artifacts. Similarly, the presence of possible air bubbles in the membranes might also induce such artifacts.

The fraction of artifacts did not increase upon the insertion of the generator into the culture medium, switching on the gas flow, or building the gradient, suggesting that it did not lead to measurable deterioration of electrophysiological signals. It is important to refer that more than half of the artifacts were detected based on bigger peaks in vicinity which may result from neurons firing almost synchronously near the same electrode or noisy action potential peaks that would be counted in double if considered. In addition, mean values of noise estimates that were used for event detection was also hardly affected by insertion and usage of the gradient generator, and mean waveforms were consistent throughout all configurations. Together, these findings show that the oxygen gradient generator did not interfere with electrophysiological recording.

### Variations in firing rates

Firing rates showed some variation between both cultures, and between the different configurations used. Analysis of normalized firing rates allowed for assessment of relative changes in firing rate in response to connecting the gradient generator and imposing flows through the inserts (Figure 4E). In both cultures, there was a small decrease when the gradient lid was sealed with no gas flow through the inserts. This may be related to oxygen availability, possibly due to the gradient generator forming a barrier for oxygen diffusion from the environment around the culture chamber to the culture. In both cultures firing rates reached a minimum when the oxygen gradient was on, probably reflecting the further reduced availability of oxygen. Activity in cultured neuronal networks has been shown to decrease with lower oxygen availability (Le Feber et al., 2017). This idea is supported by the observation that firing rates partially recovered when oxygen was resupplied through both inserts.

### Possible effect of the gradient and future work

Data presented here were collected to study the applicability of the gradient generator for electrophysiological recordings from neuronal cultures, and do not yet support conclusions on the effect of the oxygen gradient on neuronal networks. However, the oxygen gradient seemed to affect the firing rates at some electrodes, which appeared to be reversible upon reoxygenation. Nevertheless, the extent and dynamics of this influence remains to be studied and characterized.

Using the oxygen gradient generator to mimic the interaction between neurons in the penumbra and in surrounding healthy tissue, may lead to a deeper knowledge of how network activity and neuronal viability are affected, as well as the interaction between them.

## Conclusion

In conclusion, the developed device was shown to create a robust oxygen gradient within an interval of 30 min across neuronal cultures on multi electrode arrays. This gradient was well-reproducible with no transverse component and covered a length of 3.5 mm which is near the spanning area of the multielectrode arrays used. The oxygen gradient generator is cytocompatible with neuronal cultures and introduces no major artifacts or noise, making it a useful tool for the *in vitro* study of ischemic stroke.

This study provides a proof of principle that the developed generator is able to reliably impose an oxygen gradient on neuronal cultures without biasing neuronal viability or electrophysiological recording and may thus be used to enrich *in vitro* stroke models.

## Data availability statement

The raw data supporting the conclusions of this article will be made available by the authors, without undue reservation. 3D design specifications of the developed device can be made available upon reasonable request to the authors.

## Ethics statement

The animal study was reviewed and approved by the Dutch Committee on Animal Use (Centrale Commissie Dierproeven); AVD110002016802.

## Author contributions

JF conceived the study design. JK and PK designed and constructed the generator. EB enabled oxygen sensing. JS wrote the analysis software and conducted the experiments. JS and JF analyzed the results. JS, JF, and JK wrote the original draft. All authors reviewed the manuscript and approved the submitted version.

## References

[B1] Boal D. (2012). *Mechanics of the cell*, 2nd Edn. Cambridge, MA: Cambridge University Press. 10.1017/CBO9781139022217

[B2] Bossink E. G. Slob J. V. Wasserberg D. Segerink L. I. Odijk M. (2020). *Versatile fabrication and integration method of optical oxygen sensors in organ-on-chips.* Rotterdam: 2020 IEEE SENSORS, 1–4. 10.1109/SENSORS47125.2020.9278735

[B3] Boyce M. W. Simke W. C. Kenney R. M. Lockett M. R. (2018). Generating linear oxygen gradients across 3D cell cultures with block-layered oxygen controlled chips (BLOCCs) microelectron. *Anal. Methods* 195 107–113. 10.1039/C9AY01690B PMC7079814 32190125

[B4] Gaballah M. Penttinen K. Kreutzer J. Mäki A. Kallio P. Aalto-Setälä K. (2022). Cardiac ischemia on-a-chip: Antiarrhythmic effect of levosimendan on ischemic human-induced pluripotent stem cell-derived cardiomyocytes. *Cells* 11:1045. 10.3390/cells11061045 35326497 PMC8947267

[B5] Gehlen M. H. (2020). The centenary of the Stern-Volmer equation of fluorescence quenching: From the single line plot to the SV quenching map. *J. Photochem. Photobiol. C Photochem. Rev.* 42:100338. 10.1016/j.jphotochemrev.2019.100338

[B6] Hakim A. M. (1987). The cerebral ischemic penumbra. *Can. J. Neurol. Sci.* 14 557–559. 2446732

[B7] Häkli M. Kreutzer J. Mäki A. J. Välimäki H. Cherian R. M. Kallio P. (2022). Electrophysiological changes of human-induced pluripotent stem cell-derived cardiomyocytes during acute hypoxia and reoxygenation. *Stem Cells Int.* 2022:9438281. 10.1155/2022/9438281 36579142 PMC9792238

[B8] Häkli M. Kreutzer J. Mäki A. J. Välimäki H. Lappi H. Huhtala H. (2021). Human induced pluripotent stem cell-based platform for modeling cardiac ischemia. *Sci. Rep.* 11:4153. 10.1038/s41598-021-83740-w 33603154 PMC7893031

[B9] Holloway P. M. Gavins F. N. (2016). Modeling ischemic stroke in vitro: The status quo and future perspectives. *Stroke* 47:561. 10.1161/STROKEAHA.115.011932 26742797 PMC4729600

[B10] Hottowy P. Skoczeń A. Gunning D. E. Kachiguine S. Mathieson K. Sher A. (2012). Properties and application of a multichannel integrated circuit for low-artifact, patterned electrical stimulation of neural tissue. *J. Neural Eng.* 9:066005. 10.1088/1741-2560/9/6/066005 23160018 PMC3551622

[B11] Khan D. H. Roberts S. A. Cressman J. R. Agrawal N. (2017). Rapid generation and detection of biomimetic oxygen concentration gradients in vitro. *Sci. Rep.* 7:13487. 10.1038/s41598-017-13886-z 29044222 PMC5647399

[B12] Khazipov R. Congar P. Ben-Ari Y. (1995). Hippocampal CA1 lacunosum-moleculare interneurons: Comparison of effects of anoxia on excitatory and inhibitory postsynaptic currents. *J. Neurophysiol.* 74 2138–2149. 10.1152/jn.1995.74.5.2138 8592202

[B13] Lakhan S. E. Kirchgessner A. Hofer M. (2009). Inflammatory mechanisms in ischemic stroke: Therapeutic approaches. *J. Transl. Med.* 7 1–11. 10.1186/1479-5876-7-97 19919699 PMC2780998

[B14] Le Feber J. Erkamp N. Van Putten M. J. Hofmeijer J. (2017). Loss and recovery of functional connectivity in cultured cortical networks exposed to hypoxia. *J. Neurophysiol.* 118 394–403. 10.1152/jn.00098.2017 28424292 PMC5501920

[B15] Le Feber J. Pavlidou S. T. Erkamp N. Van Putten M. J. Hofmeijer J. (2016). Progression of neuronal damage in an in vitro model of the ischemic penumbra. *PLoS One* 11:e0147231. 10.1371/journal.pone.0147231 26871437 PMC4752264

[B16] le Feber J. Rutten W. L. Stegenga J. Wolters P. S. Ramakers G. J. van Pelt J. (2007). Conditional firing probabilities in cultured neuronal networks: A stable underlying structure in widely varying spontaneous activity patterns. *J. Neural Eng.* 4 54–67. 10.1088/1741-2560/4/2/006 17409480

[B17] Lee S. K. Okura I. (1997). Photostable optical oxygen sensing material: Platinum tetrakis(pentafluorophenyl)porphyrin immobilized in polystyrene. *Anal. Commun.* 34 185–188. 10.1039/a701130j

[B18] Mauleon G. Fall C. P. Eddington D. T. (2012). Precise spatial and temporal control of oxygen within in vitro brain slices via microfluidic gas channels. *PLoS One* 7:e43309. 10.1371/journal.pone.0043309 22905255 PMC3419219

[B19] Metsälä O. Kreutzer J. Högel H. Miikkulainen P. Kallio P. Jaakkola P. M. (2018). Transportable system enabling multiple irradiation studies under simultaneous hypoxia in vitro. *Radiat. Oncol.* 13 1–11. 10.1186/s13014-018-1169-9 30424810 PMC6234660

[B20] Muzzi L. Hassink G. Levers M. Jansman M. Frega M. Hofmeijer J. (2017). Mild stimulation improves neuronal survival in an in vitro model of the ischemic penumbra. *J. Neural Eng.* 17:016001. 10.1088/1741-2552/ab51d4 31658455

[B21] Orcheston-Findlay L. Hashemi A. Garrill A. Nock V. (2020). A microfluidic gradient generator to simulate the oxygen microenvironment in cancer cell culture. *Anal. Method* 12 18–24. 29123197

[B22] Pires Monteiro S. Voogd E. Muzzi L. De Vecchis G. Mossink B. Levers M. (2021). Neuroprotective effect of hypoxic preconditioning and neuronal activation in a in vitro human model of the ischemic penumbra. *J. Neural Eng.* 18:036016. 10.1088/1741-2552/abe68a 33724235

[B23] Place T. L. Domann F. E. Case A. J. (2017). Limitations of oxygen delivery to cells in culture: An underappreciated problem in basic and translational research. *Free Radic. Biol. Med.* 113 311–322. 10.1016/j.freeradbiomed.2017.10.003 29032224 PMC5699948

[B24] Rexius-Hall M. L. Rehman J. Eddington D. T. (2017). A microfluidic oxygen gradient demonstrates differential activation of the hypoxia-regulated transcription factors HIF-1α and HIF-2α. *Integr. Biol.* 9 742–750. 10.1039/C7IB00099E 28840922 PMC5603417

[B25] Romijn H. J. van Huizen F. Wolters P. S. (1984). Towards an improved serum-free, chemically defined medium for long-term culturing of cerebral cortex tissue. *Neurosci. Biobehav. Rev.* 8 301–334. 10.1016/0149-7634(84)90055-1 6504415

[B26] Shaw F. Z. Chen R. F. Tsao H. W. Yen C. T. (1999). A multichannel system for recording and analysis of cortical field potentials in freely moving rats. *J. Neurosci. Methods* 88 33–43. 10.1016/S0165-0270(99)00010-2 10379577

[B27] Stegenga J. Le Feber J. Marani E. Rutten W. L. (2008). Analysis of cultured neuronal networks using intraburst firing characteristics. *IEEE Trans. Biomed. Eng.* 55 1382–1390. 10.1109/TBME.2007.913987 18390329

[B28] Sun M. K. Xu H. Alkon D. L. (2002). Pharmacological protection of synaptic function, spatial learning, and memory from transient hypoxia in rats. *J. Pharmacol. Exp. Ther.* 300 408–416. 10.1124/jpet.300.2.408 11805198

[B29] Taxis Di Bordonia E. Valnigra D. Hassink G. C. Levers M. R. Frega M. Hofmeijer J. (2022). The association between hypoxia-induced low activity and apoptosis strongly resembles that between TTX-induced silencing and apoptosis. *Int. J. Mol. Sci.* 23:2754. 10.3390/ijms23052754 35269895 PMC8911517

[B30] Tornberg K. Välimäki H. Valaskivi S. Mäki A. J. Jokinen M. Kreutzer J. (2022). Compartmentalized organ-on-a-chip structure for spatiotemporal control of oxygen microenvironments. *Biomed. Microdevices* 24 1–11. 10.1007/s10544-022-00634-y 36269438 PMC9587069

[B31] Uchida H. Sato A. Miyayama A. Tsukada K. (2013). Generation of an oxygen gradient in a microfluidic device and cellular analysis in hypoxia. *Adv. Biomed. Eng.* 2 143–149. 10.14326/abe.2.143

[B32] Välimäki H. Hyvärinen T. Leivo J. Iftikhar H. Pekkanen-Mattila M. Rajan D. K. (2020). Covalent immobilization of luminescent oxygen indicators reduces cytotoxicity. *Biomed. Microdevices* 22 1–12. 10.1007/s10544-020-00495-3 32494857 PMC7270993

[B33] Wagenaar D. Demarse T. B. Potter S. M. (2005). “MeaBench: A toolset for multi-electrode data acquisition and on-line analysis,” in *Proceedings of the 2nd international IEEE EMBS conference on neural engineering*, (Arlington, VA), 518–521. 10.1109/CNE.2005.1419673

